# Supplementary material for: Imbalanced classification for protein subcellular localization with multilabel oversampling
Source: Bioinformatics. 2022 Dec 29;39(1):btac841. doi: 10.1093/bioinformatics/btac841 (PMC9825308; doi:10.1093/bioinformatics/btac841)
Supplement: btac841_Supplementary_Data [file btac841_supplementary_data.pdf]

Bioinformatics
doi.10.1093/bioinformatics/xxxxxx
Advance Access Publication Date: Day Month Year
Manuscript Category

OXFORD

Bioimage informatics

Imbalanced classification for protein subcellular localisation with multilabel oversampling

Priyanka Rana, Arcot Sowmya, Erik Meijering, Yang Song *

[1] School of Computer Science and Engineering, University of New South Wales, Sydney, NSW 2052, Australia

*To whom correspondence should be addressed.

Associate Editor: XXXXXXX

Received on XXXXX; revised on XXXXX; accepted on XXXXX

Contact: yang.song1@unsw.edu.au

1 Supplementary tables and figures

Table 1. Class details with its corresponding imbalance ratio per label value

Minority/Medium/Majority	Class number (C)	Subcellular location	Imbalance ratio per label
**Minority**	27	Rods and rings	1171.36
**Minority**	15	Microtubule ends	613.57
**Minority**	10	Lysosomes	460.18
**Minority**	9	Endosomes	286.33
**Minority**	8	Peroxisomes	243.11
Medium	20	Lipid droplets	74.91
Medium	17	Mitotic spindle	40.02
Medium	24	Aggresome	39.28
Medium	26	Cytoplasmic bodies	24.31
Medium	16	Cytokinetic bridge	24.0
Medium	13	Focal adhesion sites	18.73
Medium	12	Actin filaments	16.07
Medium	22	Cell junctions	14.29
Medium	18	Microtubule organizing center	12.80
Medium	6	Endoplasmic reticulum	12.0
Majority	11	Intermediate filaments	11.80
Majority	14	Microtubules	11.77
Majority	1	Nuclear membrane	10.28
Majority	19	Centrosome	8.69
Majority	3	Nucleoli fibrillar center	8.25
Majority	4	Nuclear speckles	6.93
Majority	5	Nuclear bodies	5.13
Majority	7	Golgi apparatus	4.56
Majority	23	Mitochondria	4.35
Majority	2	Nucleoli	3.556
Majority	21	Plasma membrane	3.41
Majority	25	Cytosol	1.57
Majority	0	Nucleoplasm	1

© The Author 2015. Published by Oxford University Press. All rights reserved. For permissions, please e-mail: journals.permissions@oup.com

Table 2. Improvement in F1-scores of medium classes after oversampling

	**F1-score**		
Class Number (C)	Base Model	Proposed method	%age increase
20	0.48	0.63	31.3
17	0.58	0.64	10.3
24	0.74	0.80	8.1
26	0.48	0.54	12.5
16	0.50	0.54	8.0
13	0.67	0.70	4.5
12	0.71	0.74	4.2
22	0.65	0.68	4.6
18	0.63	0.65	3.2
6	0.65	0.68	4.6
	0.61	0.66	

.

Table 3. F1-score for all individual classes in TS1. Best performance per class and per method is indicated in bold.

Class Number	Minority/Medium/Majority	Proposed method	Base model	Focal-Lovász	SIFLoc	Metric learning
0	Majority	**0.87**	0.83	0.84	0.81	0.69
1	Majority	**0.85**	0.84	0.84	0.79	0.64
2	Majority	**0.82**	0.80	0.81	0.66	0.47
3	Majority	**0.78**	0.77	0.77	0.71	0.61
4	Majority	**0.81**	0.79	0.80	0.70	0.58
5	Majority	**0.72**	0.70	0.71	0.45	0.47
6	Medium	**0.68**	0.65	0.64	0.38	0.29
7	Majority	**0.84**	0.83	0.83	0.62	0.51
8	Minority	**0.82**	0.62	0.71	0.57	0.20
9	Minority	**0.88**	0.75	0.87	0.67	0.16
10	Minority	**1.0**	0.70	0.91	0.62	0.0
11	Majority	**0.77**	0.74	0.76	0.46	0.58
12	Medium	**0.74**	0.71	0.73	0.48	0.19
13	Medium	**0.70**	0.67	0.68	0.49	0.14
14	Majority	**0.86**	0.85	**0.86**	0.43	0.57
15	Minority	**0.57**	0.0	0.0	0.46	0.0
16	Medium	**0.54**	0.50	0.53	0.45	0.29
17	Medium	**0.64**	0.58	0.61	0.61	0.14
18	Medium	**0.65**	0.63	0.62	0.10	0.13
19	Majority	**0.69**	0.68	0.67	0.44	0.46
20	Medium	**0.63**	0.48	0.62	0.08	0.0
21	Majority	**0.71**	0.69	0.69	0.39	0.47
22	Medium	**0.68**	0.65	0.65	0.31	0.17
23	Majority	**0.80**	**0.80**	0.79	0.36	0.38
24	Medium	**0.80**	0.74	0.76	0.0	0.15
25	Majority	**0.69**	0.65	0.67	0.27	0.56
26	Medium	**0.54**	0.48	0.53	0.0	0.0
27	Minority	**0.67**	0.0	0.0	0.0	0.0
		**0.74**	0.65	0.68	0.44	0.32

.

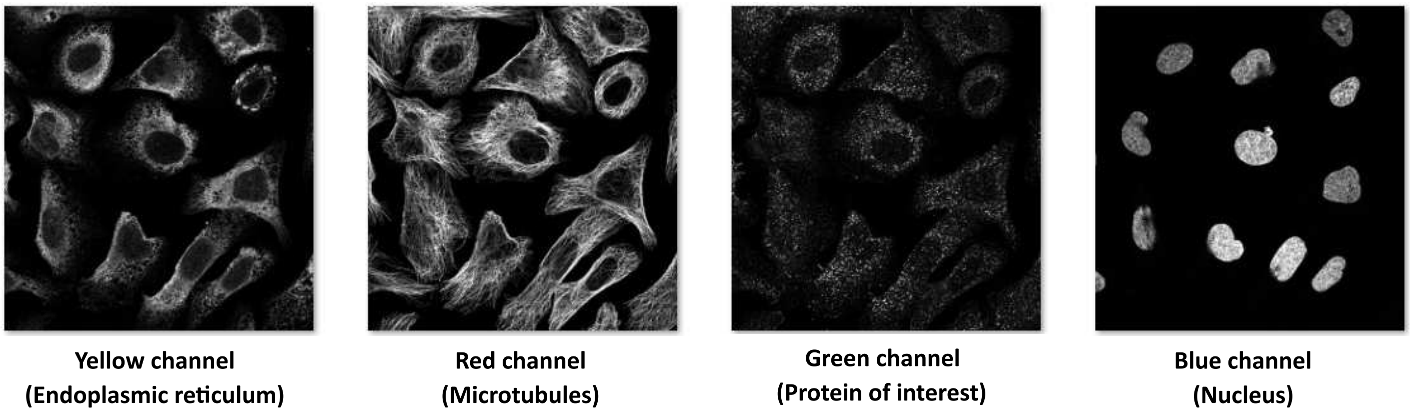

Fig. 1. Each sample consists of four channels: yellow, red, green and blue representing endoplasmic reticulum, microtubules, protein of interest (POI) and nucleus respectively. In this figure POI is class 8 which is Peroxisomes.

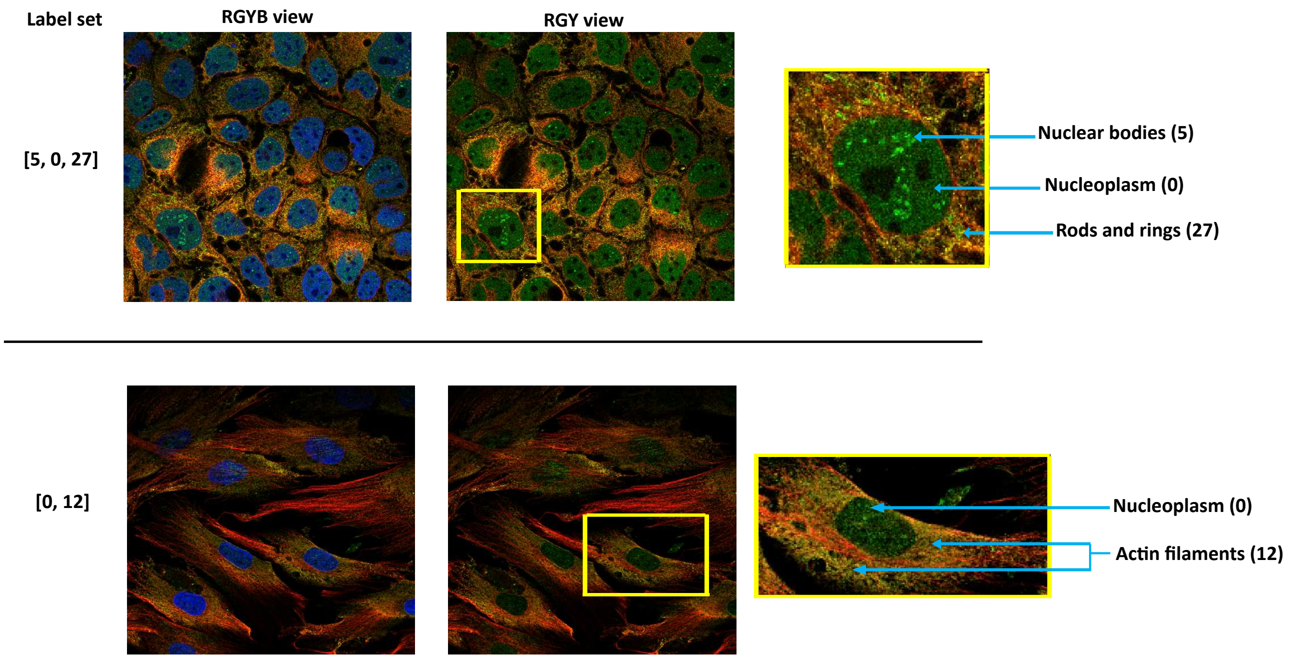

Fig. 2. Sample images showing multilocalised protein patterns in different cell lines that makes the protein subcellular localisation task challenging.

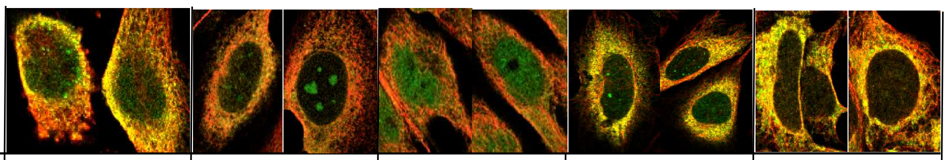

Original label set		[0,8]	[2,7,8]	[0,15]	[0,5,15]	[23,27]
Predicted label set	Our method	[0,8]	[2,7,8]	[0,15]	[0,5,15]	[23,27]
	Wang, 2020	[0,7]	[2,7]	[0]	[5]	[23]

Fig. 3. Minority class sample images from TS1 demonstrate better performance of our method than Focal + Lovász loss (Wang, 2020).

2 Details of applied metric learning

In metric learning, the definition of the positive and negative pairing is crucial, to make the model learn to distinguish between images. However, it is not straightforward in the multilabel environment. We explored triplet margin loss function (Ding *et al.*, 2015) with weighted sampling to evaluate metric learning, as unlike contrastive loss, triplet margin loss is able to tolerate some intraclass variance. Similar to contrastive loss function, the triplet margin loss function also aims to project similar images nearby and dissimilar images far away from each other. In order to achieve that, triplet margin loss builds triplets, which constitutes an anchor image, a positive image which is of the same class as the anchor, and a negative image which is of a different class from the anchor. Since protein subcellular localisation is a multilabel classification task of protein images with more than 500 different label sets and high intraclass variance due to images from different phenotypes, we applied overlapping (an estimate of the number of common labels) of label sets as a criterion to mine the positive/negative pairs. In a minibatch, the image which has the maximum overlap of labels with the anchor image is considered positive, while the image pairs with the number of common labels as zero (specifically for minority classes) or one are considered as negative pairings.

Hyperparameter margin for triplet margin loss was set to 0.5.

References

Ding, S. *et al.* (2015). Deep feature learning with relative distance comparison for person re-identification. *Pattern Recognition*, **48**(10), 2993–3003.

Wang, X. (2020). Human protein classification in microscope images using deep learning and Focal-Lovász loss. In *2020 The 4th International Conference on Video and Image Processing*, pages 230–235.
